# Charcot redux

**DOI:** 10.1093/brain/awaf386

**Published:** 2025-10-14

**Authors:** A J Lees

**Affiliations:** The National Hospital, Queen Square London, London WC1N 3BG, UK

## Abstract

Jean-Martin Charcot was born in Paris on 29 November 1825. To mark the bicentenary of his birth, the International Society for the History of the Neurosciences held a meeting in July 2025 at the Brain Institute in Paris. AJ Lees discusses the event and reflects on how Charcot’s scientific legacy continues to shape neurology today.

***How Charcot’s scientific legacy continues to shape neurology.***

Jean-Martin Charcot was born in Paris on the 29th November 1825. To celebrate the bicentenary of his birth, a meeting of the International Society for the History of the Neurosciences took place between 1 and 5 July 2025 at the Brain Institute in Paris. The 5-day meeting was attended by neurologists, medical historians and psychologists and was chaired by the distinguished Charcot scholar, Doctor Olivier Walusinski. The 41 oral presentations were divided into seven separate 3–4-h sessions that covered Charcot’s clinical and pathological contributions, his art, and his lasting influence on French culture. There was a poster exhibition outside the amphitheatre which included topics as varied as descriptions of Charcot’s shower and his enthusiasm for hydrotherapy, and correspondence between Charcot and Cláudio Velho da Motta Maia, Dom Pedro II, the Emperor of Brazil’s private physician.

Many delegates took the opportunity to visit the Charcot Library and view Charcot’s papers, working notes, and drafts of his lectures and sketches. A historical tour of the Salpêtrière took place on the Thursday morning with a focus on the sector where Charcot had worked for most of his professional life and the hospital chapel of Saint-Louis, where his funeral mass had taken place. We were also shown the niche in the wall at the entrance of the hospital where his bronze statue had stood from 1898 until its melting down during the Nazi occupation of Paris. An excursion to the old Faculty of Medicine provided delegates with the opportunity to stand under *Une Leçon Clinique a La Salpêtrière*, the famous tableau by André Brouillet and view the historical treasures in the Museum of the History of Medicine. There was also a visit to Charcot’s summer residence in Neuilly-sur-Seine where his son, Jean-Baptiste Charcot, the polar explorer, was born and where the Charcot family still live. The banquet was held at Charcot’s former family home at the Hôtel de Varengeville, an 18th century mansion on the Boulevard St Germain that he had bought in 1884 (Fig. 1).

**Figure 1:**
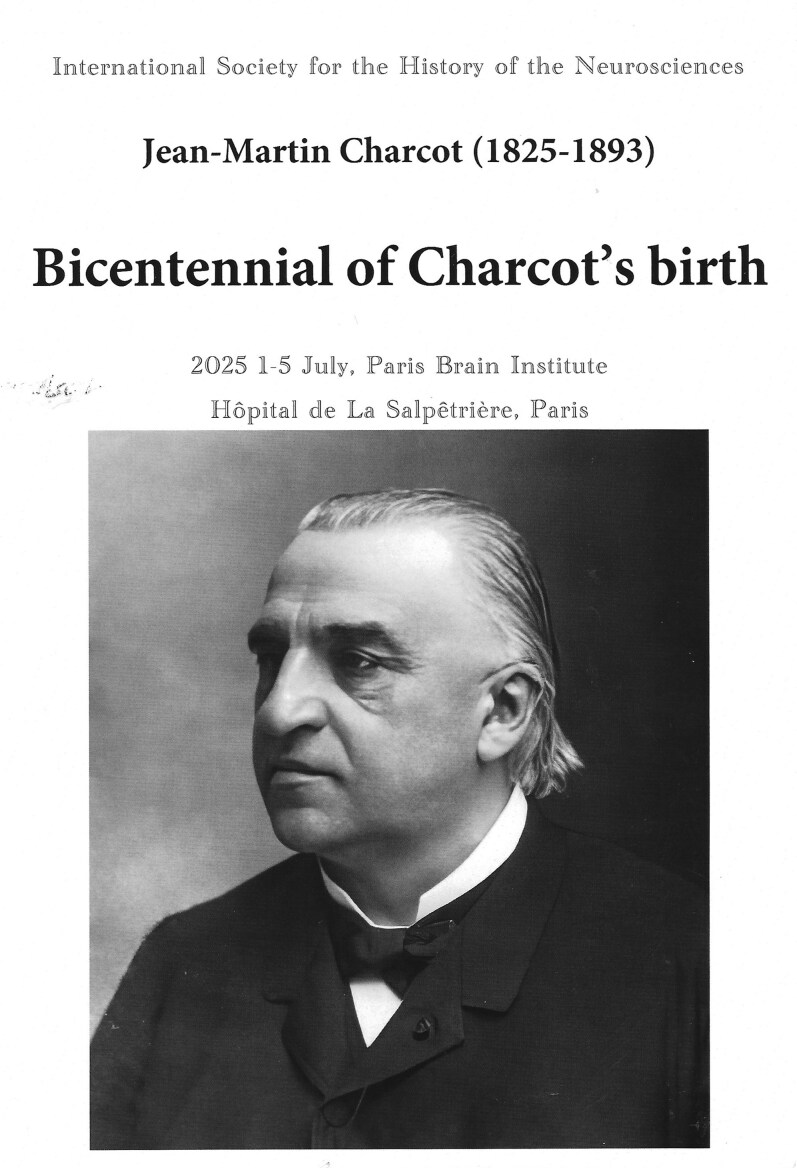
**Frontispiece for the Bicentennial of Charcot’s birth that took place between 1 and 5 July 2025 at the Brain Institute, the Salpêtrière Hospital, Paris**.

While the meeting provided ample evidence of the continuing interest in Charcot’s work, his organizational genius and his medical achievements, it also reflected changing attitudes towards the past. The centenary event of his birth had been a celebration of France as a nation, as well as a tribute to Charcot’s greatness as the founding father of neurology. A solemn service was held at the Sorbonne attended by the President of the Republic and presided over by the Minister of Education. Two of Charcot’s favourite students Joseph Babinski (1857–1932) and Pierre Marie (1853–1940) and some of the foreign guests who had worked at the Salpêtrière, such as Gheorge Marinescu (1863–1938) and Cornelis Winkler (1855–1941), delivered speeches highlighting his scientific achievements and comparing him to Louis Pasteur and Claude Bernard. Seven hundred delegates attended a formal dinner at the Palais d’Orsay, where an address of thanks was given by Charcot’s son, Commander Jean-Baptiste Charcot (1867–1936). There was also a formal reception at the Hôtel de Ville hosted by the Municipal Council of Paris with music provided by singers from the Paris Opera. Recent advances in two of Charcot’s particular interests, namely motor neuron disease and migraine were the focus at the accompanying 25th anniversary meeting of the Société Française de Neurologie held at the Salpêtrière hospital. In the past 50 years Charcot has been the object of intense scrutiny by historians, broadening scholarship beyond his neurological achievements. As a consequence, there was now no longer a uniform collective memory at the Bicentenary lectures, leading to a more balanced judgement of Charcot’s enduring importance for neurology.

## The quest

As I rotated through the different clinical specialities at medical school I learned about Charcot’s neuropathic joint, then his two triads (biliary and neurological), his crystals (Charcot-Leiden) and finally some of the neurological diseases he helped to delineate; motor neuron disease (still known as Charcot’s disease in France), hereditary peroneal muscular atrophy (Charcot-Marie-Tooth), *la sclérose en plaques* (multiple sclerosis), and the small microaneurysms found in the deep penetrating arteries of the brain (Charcot-Bouchard). Ronald Henson, one of my medical school instructors told my class that if you removed all Charcot’s original discoveries from the annals of neurology, the speciality would be unrecognizable.

After my house jobs I set off for Paris on a peregrination to understand how Jean-Martin Charcot had transformed the nosography of nervous disease in little over a decade. At the Salpêtrière hospital his name was rarely mentioned on ward rounds. When I questioned why, I usually received a Gallic shrug or a reply that his pre-eminence was taken for granted. The *chef de clinique* told me that Charcot had dented his reputation by his mistaken theories relating to the effects of hypnotism on hysteria. He also told me that Charcot’s son, Jean-Baptiste, the polar explorer, was much more widely remembered and respected.

The nine volumes of Charcot’s collected works, several of which were translated into English as *Lectures on the Diseases of the Nervous System delivered at the Salpêtrière hospital*, gave me a flavour of Charcot’s teaching method as well as his scientific contribution.^1^ Each lesson began with a summary, followed by a detailed, sometimes illustrated account of the chosen topic, liberally embellished with footnotes and references. These lessons made me appreciate the extent of Charcot’s erudition and his attention to detail. Bourneville, who collated Charcot’s lectures wrote that there was enough unpublished material for at least another six volumes. Lesson V in Volume 1 on Paralysis Agitans began:

Gentlemen: those among you who this morning passed through our wards were probably surprised to find collected there so great a number of female patients in whom tremor seems to constitute the paramount or at least the most striking symptom of the disease which they labour under. This gathering of patients, forming a genus apart I purposely contrived. In that way I desired to enable you to recognise by means of a comparative study, certain shades of distinction and even marked differences which the examination of isolated cases does not allow you to so readily discern. At first glance you may have thought the scene monotonous in character … one thing alone strikes the gazer’s glance as peculiarly noticeable; it is the diversity in position and intensity of the rhythmical oscillations of the limbs. But a more attentive inspection soon allowed you to distinguish, under this apparent uniformity, different features which at first had completely escaped you.^1^

One day Jean-Claude Gautier (1926–2013), one of my teachers at the Salpêtrière suggested I also read *Les Leçons du Mardi*, two thick volumes made up of 49 separate dialogues between Charcot and his patients from 1887 to 1888. In one lesson Charcot told his students:

I am not of the type to suggest things that cannot be demonstrated experimentally. You know that, as a principle, I pay little attention to abstractions and have no use for preconceived notions. If you want to see clearly, you must take things exactly as they are … . What a marvel it would be if I could, in fact fabricate illnesses according to my whims and fantasies. But in fact all I am is a photographer.^1^

In some of the 200 or so transcripts where Charcot had spoken directly to his assistants rather than the patient, I detected a geniality that belied his reputation as an austere ambitious autocrat; there were also occasional flashes of dry humour. In the most desperate cases he seemed reluctant to declare there was no hope. The more serious the patient’s malady, the more kindly and attentive he came over. He never used frightening words in front of patients and publicly, at least, he was reluctant to attempt to analyse a patient’s subconscious mind. He told his students there were things that were better left hidden. Sometimes in a consolatory tone he would quote from Greek tragedy or Shakespeare:

What have we done, O Zeus to deserve this destiny?

Our fathers were wanting, but we, what have we done?

The Tuesday lessons gave me a better appreciation of Charcot’s manner in the classroom. He always gave primacy to the clinical gaze and observation was always his starting point for diagnosis. Recognition led to the imprinting of an image in his brain, which he was then able to convert into action. He was a master of defamiliarization. As Foucault later wrote in the clinic it was the ‘eye that knows and decides, the eye that governs’. Charcot’s *modus operandi* seemed much closer to that used by a criminal detective than a thaumaturge:

Imagination, left to its own devices, abandons itself to unrealizable dreams; science holds it back and teaches us what cannot be. It does not follow that science contains the principle of art, but rather that we must study science either before or at the same time as art, in order to learn the limits by which art must be contained.^2^

As I strolled past the pharmacy where Charcot’s consulting room had once stood and then sauntered between the rows of decaying chalets where some of the elderly and infirm women under his care had resided, I was caught up in a drama. Some of his words I had read in the Tuesday lessons kept coming back. There were flashbulb moments when I felt I was almost in touching distance.

## The method

Charcot’s father, Simon-Pierre, a carriage maker and member of the Parisian petit-bourgeoisie, believed it was his duty to select and recommend professions for his children. One day he said to the oldest of his four sons, ‘As for you Jean, because you have demonstrated so much aptitude for drawing you will become an artist, but if you prefer, because you are also studious you can complete your higher education and become a physician.'

After considerable deliberation Jean-Martin Charcot opted for medicine but he intermixed his love for art and painting with his practice to such an extent that his clinical assistant Henri Meige wrote, ‘Charcot the physician is inseparable from Charcot the artist.' Meige believed that Charcot had an artist’s eye and it was this which allowed him to decipher the contours of disease and describe the elements of its expression. Sigmund Freud, who spent a few months at the Salpêtrière, believed Charcot also had the nature of an artist, noting that he would sometimes include a pen sketch of a patient if the written description was becoming too verbose.

In 1853, Charcot defended his inaugural thesis on the clinical distinction between chronic gouty arthropathy, rheumatoid disease and osteoarthrosis. His dissertation included original research carried out while he had been an intern at the Salpêtrière hospital, and his clinical descriptions and distinctions were embellished with some of his own sketches. His examiners were particularly impressed by the fact that he had not only comprehensively reviewed the French medical literature but had also read and referenced important contributions by foreign authors written in English and German. The jury awarded him the highest distinction. Charcot’s thesis was the blueprint for a method of enquiry that would lead him to delineate previously unrecognized neurological disorders after his eventual return to the Salpêtrière hospital in 1862.

In spite of this promising start to his medical life Charcot’s early career was undistinguished, and his advancement through the Parisian hospital system was slow and gradual. While waiting in a long line for a tenured appointment he did a great deal of work in polyclinics rotating through various medical specialities. During this uncertain time he had the good fortune to come under the patronage of Pierre Rayer (1793–1867) at La Charité hospital. Rayer became his mentor and also provided badly needed financial assistance by recommending Charcot to a rich family in search of a personal physician. Rayer also facilitated Charcot’s membership of *La Société de Biologie* thus allowing him to attend and present research findings to an august learned society, presided over by Rayer and the physiologist Claude Bernard (1813–78). The audience was made up of lively young scientists including medical contemporaries like Charles Édouard Brown-Séquard (1817–94), Alfred Vulpian (1826–87) and Charles-Phillipe Robin (1821–85). Charcot also started to attend the weekly meetings of *La Société anatomique de Paris* formed by Laennec and Dupuytren in 1803 but disbanded 5 years later after an acrimonious quarrel relating to plagiarism. The society was reconstituted in 1826 under the auspices of Jean Cruveilhier (1791–1874), the first holder of the chair of pathological anatomy at the Paris faculty of medicine. Cruveilhier’s pathological findings in multiple sclerosis and progressive muscular atrophy would provide building blocks for Charcot to later establish these conditions as distinct nosological entities.

When Charcot eventually became the chief of the Cazalis service at the Salpêtrière and decided with his friend Vulpian to examine every one of the thousands of patients under their care, the vast size of the hospice was a deterrent and Charcot soon limited his visits to the wards. Instead he preferred to see the patients in his office and later, after the opening of the polyclinic in 1880, in a room in the outpatients department. On 3 days of the week he would arrive at the clinic at 09:30 a.m. and sit behind a small railing separating him on one side from the patient, and on the other from the students.

**Figure 2:**
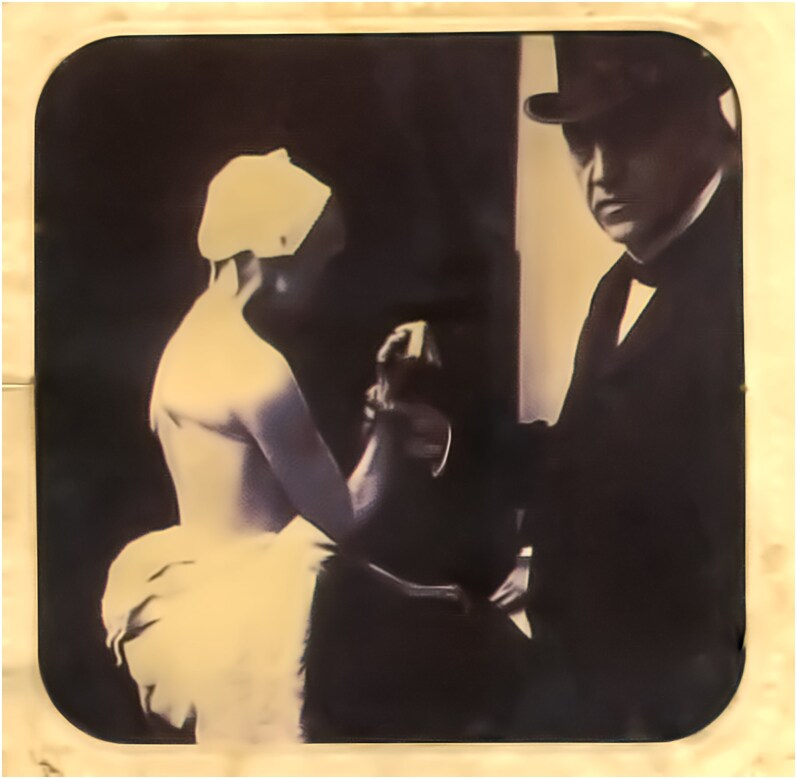
**Photographic print (*papier albuminé 71 × 82 mm collé sur carton*) showing Jean-Martin Charcot examining Madame Streilh, a patient with locomotor ataxia**. From the private collection of Dr Olivier Walusinski.

It is often supposed and written that Charcot was a ‘no touch’ physician who relied on close inspection of the naked patient and the medical history presented by his junior staff to make a diagnosis. In fact there is ample evidence that when needed he would carry out almost all the elements that comprise the modern neurological examination. He tested visual acuity and visual fields, specifically looked for abnormalities of eye movements and the presence of nystagmus and was an early convert to the value of the ophthalmoscope. When testing facial weakness, he was aware of Charles Bell’s distinction between a peripheral and a central lesion. He evaluated hearing with a ticking pocket watch placed at different distances from the patient’s ear. The Skoda rubber-ended hammer that had been first developed as a percussion instrument was used to test the knee jerk and he routinely looked for the presence of primitive reflexes. Muscle power was assessed by observing the patient carrying out voluntary movements including rising from a chair and elevating the arms (Fig. 2).

Sometimes he used a dynamometer for testing grip strength. Muscular atrophy was assessed by measuring the girth of a limb with a measure. To test pain he would pinch or prick the skin, and he devised a thermometer that could be used to safely evaluate temperature sensation without causing skin burns. He used Romberg’s test to evaluate the many cases of sensory and locomotor ataxia. The gait was always carefully evaluated and he had a small gait laboratory, where his assistant Gilles de la Tourette (1857–1904) would assess the length and width of stride using Indian ink to record footprints on rolls of paper.

His longstanding interest in the controversies related to cerebral localisation led him to carry out detailed evaluations of language, reading, writing and calculation. Charcot possessed the best equipped medical photographic laboratory in Europe but despite his appreciation of its value in documenting physical signs and providing a visual record of a patient’s abnormalities at different stages of disease, photographs never replaced his drawings and sketches in his own publications. It would be the generation of physicians that came after Charcot that would turn the neurological examination into a systematic scientific experiment by incorporating the growing number of anatomo-clinical correlations into a new semiology. The *phenomène des orteils*, for example, that is still so valuable in distinguishing an upper motor neuron lesion in the corticospinal pathway from the various causes of neuromuscular weakness and psychogenic paresis was reported by Joseph Babinski 3 years after his teacher’s death.

Charcot also cut open cadavers in a renovated kitchen behind his consulting room. If his lancet revealed an alteration in the colour, volume or texture of the brain he sketched the abnormality in a notebook and then included the coloured drawing with his written post-mortem findings in the medical dossier. The autopsy was an integral extension of Charcot’s clinical examinations carried out in life, which allowed him to make connections between symptoms and pathological lesions. These clinico-pathological correlations created a new system of neurological semiotics. He also believed that the autopsy was the final service one could offer the deceased patient’s family.

Structural disorders of the peripheral and central nervous system with the exception of some higher cortical centres became Charcot’s area of expertise. Most of the maladies that he took a particular interest in, manifested external visible signs. Some such as motor neuron disease had pathological abnormalities in the brain and spinal cord, which correlated with the clinical picture giving them a structural solidity of the sort found in many general medical disorders. Others like epilepsy, chorea and Parkinson’s disease that he classed as ‘nevroses’, had no identifiable consistent pathology but Charcot was convinced that they would yield their secrets to histological advances in the future. The boundaries he created in his hospital practice later came to define the speciality of neurology and distinguish it from asylum medicine. Hysteria, another neurosis, also presented with florid neurological signs and became an all-consuming later interest. In the first 9 months of 1891, Charcot had 3148 consultations and 1913 new referrals, of whom 806 were listed as hysteria or nevroses. Achille Souques, who had done his training at the Salpêtrière, speculated that Charcot may have chosen nervous disorders as his area of speciality because of the unavoidable visibility of much of its symptomatology.^3^ In order to allow him to cope with his large practice he routinely asked his assistants at the hospital and in private practice to take the history and carry out the examination.

Only about 10% of patients seen in Charcot’s hospital clinics and less than 1% of his publications would be considered to fall within the modern speciality of psychiatry but in private practice Charcot saw many patients with mental illness. His practice was advertised in the newspapers as, ‘Dr. Charcot nervous and mental diseases, Mondays, Wednesdays, Fridays from 3 to 6 PM, 217 Boulevard St-Germain.’ Some of the patients he saw with neurasthenia, psychosexual neuroses and hysteria were admitted under his care to the Institut Hydrotherapique de Passy-Paris, a magnificent establishment on the heights of the Trocadero, with a garden of 8000 m. As well as separating them from a pathological family environment, his patients received intensive treatment including the use of water jets and sulphur baths. Some of his wealthy patients were advised to visit spas outside Paris, especially Lamalou-les-Bains in the Languedoc. On his death certificate Charcot was referred to imprecisely as an alienist.^3^

## The class

The Tuesday lessons for his assistants and medical visitors took place in a room in the polyclinic next to the dispensary. Most of Charcot’s teaching was done on outpatients allowing him to demonstrate common disorders and providing him with ample occasion to remind his students that it was often impossible to make a firm diagnosis at first clinical presentation. His method depended on the continuous study of a nervous disorder from its inception through its entire course until death.

Dogmatic teaching, or what we call ex cathedra lessons, is something artificial … My aim is to fool no one, and so before your eyes, I will plunge right in and proceed just as I do in my own practice. I … interview patients whom I don’t know.^1^

In contrast to the Friday lectures, here he was free to speculate on possible causation, informally discuss recent scientific findings and embroider his teaching with light-hearted stories and anecdotes. Visitors were often struck by the penetrative force of his impassive gaze and reported that there were many times when a single sharp glance seemed sufficient for him to make an instant diagnosis. Dr Jane Henderson, a Scottish physician, went to Paris in the autumn of 1892 after working as assistant medical officer at the Holloway Sanatorium for the Insane. Despite being female and without formal invitation she managed to attend the Tuesday demonstrations without apparent difficulty. In her reminiscences published after Charcot’s death from a fatal myocardial infarction the following year, she wrote how she had been struck by how old he had appeared, how he took short fast steps, and that when he had imitated a patient with Parkinson’s disease in one of his lessons it had come over to her as a travesty of himself. Some of those close to Charcot at this time had also noticed that he was becoming more stooped and more hesitant in his speech and gait. Henderson commented that he had a quiet slightly monotonous voice and a relaxed manner and gentle sense of humour which reduced the tension in the audience. He never spoke before first presenting the patient to the class and she was impressed that no detail was too small or no fact too unimportant for him. He was fond of using English words to embellish his descriptions like ‘high stepping’ to describe the gait of locomotor ataxia and ‘egg whipping’ for choreic movements of the hands. She also marvelled at how, once he had drawn the class’s attention to a particular sign, almost like magic the elusive diagnosis came into view.^4^

## The spectacle

With support from his politically well-connected republican colleague, and former intern Désiré Magloire Bourneville, Charcot managed to secure public funds for a large amphitheatre. At his inaugural lecture on 22 November 1879, he declared:

The construction of a room for teaching within this hospital has been a dream of mine ever since I took up my duties here in 1862. From that time, I've never ceased believing that this large, noble asylum of human miseries - where several Masters of French medicine, starting with Pinel, have carried out their illustrious work - would one day become a well-organised centre for instruction and research, or in other words, a Pathological Institute.

The new lecture hall was constructed in a building in the central yard of the hospice that had formerly been the central kitchen and could hold 400 people. Charcot was the first lecturer in the Paris Faculty of Medicine to allow ‘observers’ to attend his course of lessons. Attendance was limited to visiting physicians, a few selected journalists, social commentators and public officials, and occasionally a celebrated novelist, artist or actor was granted entry. These non-medical guests came from the same well-to-do society within which the Salpêtrière physicians themselves circulated. The master classes proved to be extremely popular and attracted much attention from the press, and their preparation became a significant focus of Charcot’s week. Other physicians like Jules Bernard Luys (1828–97) at La Charité hospital also started to demonstrate patients with hysteria to the general public.

On Friday mornings the audience entered from the back of the lecture hall and walked down past the sloping tiers of wooden benches to sit on the rows of chairs. The walls were painted in red and gas lamps hung from the roof. At the front, a platform extended across the whole length of the auditorium. Robert-Fleury’s famous canvas depicting Pinel releasing the insane from their chains hung behind the stage. A few minutes before 10 a.m. the shutters were drawn on the windows and the footlights switched on. To a hush of reverence and eager anticipation Charcot entered the crowded room from a side door and walked with lowered gaze to his seat on centre stage. He was followed into the dimly lit theatre by a preordained procession of assistants and aproned junior doctors. In front of Charcot’s seat were two small tables which contained his instruments and teaching aids. The service chiefs of medical photography, ophthalmology and electrotherapy made their way to reserved chairs on the front row while his *chef de clinique*, who would assist him with the demonstration, sat on the dais among a small number of distinguished special guests. The patients who had been waiting in the ante room behind screens were the last to enter accompanied by a nurse. Charcot began with his customary: ‘Gentlemen, today …’, his low-pitched voice resounding through the silent theatre. He spoke slowly, never raised his voice and used short phrases to make his point. He usually delivered his lecture seated but sometimes he stood side on with the patient positioned slightly behind him. He rarely used rhetorical flourishes or flamboyant gestures. He was the epitome of the sober sceptical rational scientist. A visiting journalist wrote that Charcot had hammered his points into his head like a carpenter driving a nail into a large block of wood.

Charcot was renowned for his innovative early use of audio-visual teaching aids, which he used to make science understandable and memorable. There were poster stands on the stage where he displayed charts including family trees and anatomical drawings, plaster casts of deformities and photographic prints. He also used an electrically charged ‘megascope’ to project Chinese lantern slides onto a large screen. This allowed him to show images of pathological specimens. There was a central light dimmer which would be switched off before a beam of limelight was shone onto the patient. Sometimes he would walk across the stage imitating a pathological gait or resort to props such as feather topped caps to bring out titubation in a lecture on tremor. Each lesson lasted around 2 h during which six or seven patients would be presented. At the end of the lesson he handed over his handwritten notes to his *chef de clinique* for redaction and future publication.

**Figure 3:**
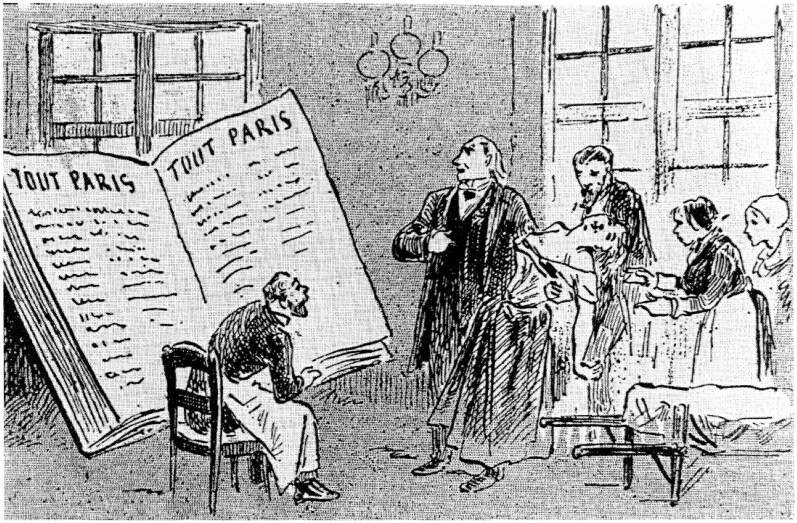
**A caricature of Brouillet’s canvas ‘*A Clinical Lesson at the Salpêtrière*’ by Draner, the sobriquet of painter and cartoonist Jules Joseph Georges Renard (1833–1926) published in *L’Univers Illustré number 1676* on 7 May 1887 and showing the cultural influence of Charcot on Paris**. From the private collection of Dr Olivier Walusinski.

The novelist and art critic, Octave Mirbeau (1848–1917) wrote that the 19th century would be viewed in history as neither the century of Victor Hugo nor of Napoleon Bonaparte, but the century of Jean-Martin Charcot. In his column in *L’Événement* he also remarked that scientific knowledge had already become a powerful weapon of control (Fig. 3):

In a few very short sentences, he poses the problem to be solved, the question to be studied, and immediately introduces the living examples. Immediately (at least that's what I felt as a newcomer) the professor fades, you no longer see him, you only hear his voice, monotonous like that of a waxwork’s showman, and your eyes are only on the deranged creatures sitting there, whom you could touch. Sometimes, at rare intervals, a bitter word, a wry smile, an eloquent quotation reminds you that there is a master here. The rest of the time, the great professor limits himself to being a demonstrator. The impression is singular at first. This figure before you is indeed a human figure and, moreover, a living one: and you hardly believe it. Seeing her in profile, moving, obediently moving a limb at Charcot's command, one might think one was watching an automaton. This figure takes on the appearance of a colored shadow puppet; her gestures are hesitant, as if poorly greased.^5^

French medicine had now started to use science and technology for the benefit of patients and Charcot’s high profile led to him being elevated to cult status (Fig. 3). Léon Daudet wrote that the Faculty of Medicine lived ‘in the radiance of his name’, not a professor was nominated, not a hospital appointment made, not a gold medal awarded without Charcot’s approval. But even at the height of his powers there were some critics. Félix Piatel, a journalist who wrote for *Le Figaro* under the pen name of Ignotus, accused him of *cabotinage* (showing-off):

He monopolised hysteria. He astonished men. He frightened women. He practised scientific overacting [cabotinage]. His success has been enormous. Oh the great allure of hamming it up! It has benefited both Charcot, and science itself. Charcot has advanced science in the manner of Richard Wagner, the great musical ham. Charcot and Wagner seem to me to be of the same tribe.^6^

Rallying to his master’s defence, Henri Meige argued that Charcot taught on all the different manifestations of nervous disease and felt that students should see examples of hysteria as part of their training:

It was an innovation. It didn't take much more for the sessions he devoted to studying the convulsive manifestations of major hysteria to be treated as theatrical exhibitions. A false, not to say malicious, insinuation, which finds its only excuse in the ignorance of those who launched it.^6^

If there was an ‘elevation’ of the spectacle then it came entirely from the patient’s presence:

As for this face, which one wanted to see as a theatrical mask, deliberately adapted to the role of a miraculous healer, is it not superfluous to recall that nature alone had created all its features? This mask was at twenty what it had been at sixty, with more dark hair and fewer wrinkles, but still modeled in the antique style, impassive, a little enigmatic, impressive. The daily razor stroke that highlighted his Caesarean profile was the only artifice added to nature.^7^

In 1882, *The Lancet* reported that there was little to distinguish the demonstrations at the Salpêtrière from the popular hypnotism cabaret shows (a viewpoint Charcot strongly resented). The anonymous correspondent also remarked that there appeared to be no counterpart in England to the grand hysterics and that medical hypnotism had never caught on in England.

At the International Congress on Physiological Psychology in 1889, Charles Richet (1850–1935) proposed that hypnosis should be distinguished from animal magnetism and reserved for a science that could be explained by suggestibility. Charcot’s view that susceptibility to hypnosis was a characteristic of hysteria and that there were three distinct stages of hypnotism—lethargy, catalepsy and somnambulism—had begun to lose out to Bernheim and Liébault’s views that it was a normal psychological phenomenon based on suggestion. A motion was also passed at the meeting to recommend the banning of all stage performances of hypnotism which met with approval from both the Salpêtrière and Nancy schools.

## Influence on Parisian culture

Charcot created a vogue for neuropathology that extended beyond the world of medical science and into the public psyche. His clinical demonstrations inspired novels, opera and modern dance. Parisian theatre too drew liberally from the raging controversies surrounding the use of medical hypnotism. After his death in 1893, criticism of his perceived theatricality and pedagogy increased. Two Judas Iscariots, Axel Munthe and Léon Daudet, openly stated in their books that he had been manipulated and tricked by ‘The Girls of the Salpêtrière’. They came to see him as a ringmaster and his patients as comical circus clowns. In *Les Morticoles* (1896), Daudet describes Charcot, thinly disguised as Foutange, as a professor of hysterics and somnambulists and also criticizes him for his unshakeable longstanding belief in hereditary social degeneration as a cause for many nervous disorders:

He does not understand this Foutange that the odious materialism that he represents, that the exploitation of man by man that science without conscience are the necessary and proximate causes of all the diseases that he tickets with baroque names and that he attributes to alcohol to syphilis, to what he calls nervous degeneration.^8^

*The Story of San Michele*, published in 1929,^9^ was recommended reading for several generations of aspiring British doctors. Munthe, a Swedish born physician attended Charcot’s lectures and wrote of his uncanny ability to get straight to the root of the problem with ‘his cold eagle eyes.’ Munthe was later banned from the Salpêtrière after attempting unsuccessfully to liberate one of Charcot’s patients and return her to live with her parents in Normandy. He went on to write many years later that Charcot had relied too much upon his eye and that his examination was dangerously superficial. Like Daudet, Munthe also believed that he had been naïve and too trusting in relation to the behaviour of the hysterics under his care. He described Charcot’s Friday lectures as ‘an absurd farce and a hopeless muddle of truth and cheating’. As an aside he added that Charcot’s saving grace was that he was indifferent to female charms and flirtations. By 1907 even Joseph Babinski, one of Charcot’s staunchest defenders, proposed that hysteria should now be replaced by a disorder he termed pithiatism that was caused by autosuggestion and could sometimes be cured by persuasion.

At the Salpêtrière, people with epilepsy and hysteria were treated alongside each other, as both conditions were considered to be functional nervous disorders. After Charcot’s death many of his well-evidenced observations on convulsive disorders were distorted, subjected to hyperbole and transformed into unscientific nonsense by music hall chancers. His lessons also acted as promotion for the emergence of a group of young female artists, nicknamed *les gommeuses epileptiques,* whose performances in the musical halls and cafes reinforced in the public imagination the ancient links between the sacred disease of epilepsy, diabolical possession and madness. The patrons of the *Moulin Rouge* went wild for the hysterical choreography of Charcot’s former patient, Jane Avril. This zeitgeist was represented in a lithograph by Edgar Degas entitled *Chanteuse Épileptique* which depicted one of the ‘epileptic singers’, Émilie Bécat with her arms in demi-bras at *Les Ambassadeurs,* an outdoor café near the Champs-Elysées. In an attempt to explore this porous membrane between the Salpêtrière hospital and the demi-monde, Georges Gilles de la Tourette attended some of the magnetizers’ shows held at fairs and in theatres in disguise. He also recruited a team of medical student ‘irregulars’ to track down and observe some of the female patients from the hospital who were moonlighting as singers and dancers.^10^

The novelist Colette (1873–1954) in her *Claudine* series of books wrote that ‘her twin’ Émilie-Marie Bouchard (1874–1939), better known to the public as Polaire (Polar Star), exhibited during her stage show all manner of electric shocks, quivers and twitches. As she sang she would twist her back into an arc, clench her fists and shake her hourglass figure like an infuriated wasp. ‘In her epilepsy’ her eyes would roll back into her head and she would sometimes fall into a swooning trance. Another ‘epileptic singer’ dubbed by the popular press as *l’epileptique sauteuse* is reported as having said, ‘When I dance I am taken over by an attack of madness that makes me forget everything, my arms are dizzy, my legs are crazy—(I am) no longer in conscious control.’ *La Parisienne épileptique,* a song made famous by Mistinguett (1873–1956) includes the words, ‘When I hear the lyrics I become epileptic’. In the view of Georges Montorgueil, editor of *Le Temps* and a friend of Gilles de la Tourette ‘at least half the popular songs of the time originated from the jiggling pit of the late Charcot … They have gesticulatory hysteria’. As well as these portrayals of epilepsy and hysteria there were references to other common nervous disorders. One chanteuse sang, ‘I’m a little woman who’s excessively nervous … In reality the blood of a turnip runs through my veins. But the fashion today, le grand chic, is to appear neurasthenic.'

Le Grand Guignol theatre opened 4 years after Charcot’s death in a small renovated Jansenist chapel in Pigalle and enjoyed popularity into the late 1930s. Its shows were designed to thrill, horrify and cause sexual arousal. In the early years some of the plays (five or six would be shown in one performance) dealt with the intrigues of the asylum, hypnosis and electrotherapy, amoral evil doctors who possessed secret dangerous knowledge, and corruption through science. During one early performance six people had fainted and several vomited, when an actress whose eyeball had been gouged out reappeared with a realistic bloody mushy hole in her skull. One of the theatre’s founders, Max Maurey reassured the general public that a ‘house doctor’ would from now be present at every performance but then went on to add that he too had fainted during one of the sensational shows.

Some of Charcot’s former assistants and colleagues became involved with the theatre. The Salpêtrière psychologist, Alfred Binet collaborated with André de Latour de Lorde, Grand Guignol’s arch dramatist of terror, in several commercially successful plays, including *Obsession* (1905), *A Lesson at the Salpêtrière* (1908), *The Horrible Experiment* (1909), *The Mysterious Man* (1910), and *Crime in a Mental Asylum* (1912). In 1901, Gilles de la Tourette, while lecturing on the medical aspects of de Lorde’s play *The Sleeper* at the Théâtre de l’Odéon had a ‘funny turn’ rendering his speech incomprehensible and providing further evidence of his deteriorating health due to general paralysis of the insane. Gilbert Ballet (1853–1916), another former Charcot *chef de clinique*, provided a preface for another Binet and de Lorde collaboration *Madness in the Theatre* (1913) and Joseph Babinski, under the *nom de plume* of Olaf collaborated with playwright Pierre Palau on a Guignol play called *The Disturbed* (1921).

André Breton (1896–1966), founder of the surrealism movement, studied medicine from 1913 to 1920 and in 1917 spent a few months working under Joseph Babinski at La Pitié hospital. One of the few to positively review *The Disturbed*, he also wrote that Babinski had attended the play’s opening night wearing a false beard. With another former medical student and poet, Louis Aragon (1897–1982), he went on to write a celebratory text, stating that hysteria had been the greatest poetic discovery of the end 19th century:

‘Hysteria is not a pathological phenomenon and can, in all respects, be considered as a supreme means of expression.’

For the surrealists, hysteria represented an emblematic display of the unconscious, a kinaesthetic imagination that permitted the psyche to appear and a template for the dramatization of the invisible. Its convulsive, aesthetic power held up a mirror to society’s embedded fears of female sexuality and madness. Breton saw Charcot’s studies on hysteria as a moving picture where science and spectacle had converged.

## Influence on British neurology

It is widely acknowledged that James Crichton Browne’s (1840–1938) decision to pursue a research programme at the West Riding Pauper Lunatic Asylum between 1866 and 1876 was an important contributor to the origins and evolution of British neurology. Crichton Browne spent time in Paris between 1862 and 1863, following in the footsteps of his father, the distinguished alienist, William Browne (1805–85), who had visited Esquirol, Pinel’s successor at the Salpêtrière hospital. His decision to develop a state-of-the-art pathological laboratory in a mental asylum and his resolve to found and edit a new journal *The West Riding Lunatic Asylum Medical Reports* are likely to have been influenced by what he had seen during his time in Paris, where the clinico-pathological method had begun to be applied to nervous disorders. One crucial distinction, however, with Charcot’s department that was also based in an asylum was Crichton-Browne’s accommodation and enthusiasm for experimental laboratory animal research including Ferrier’s early cerebral localisation experiments.

Charcot spoke good English, was familiar with the English medical literature and enjoyed reading Shakespeare. In 1861 he visited several London teaching hospitals and the Hunterian museum at the Royal College of Surgeons. A painting of Hughlings Jackson hung in his small office on the ground floor of the hospice’s Pariset division. Charcot attended the 37th British Medical Association meeting held in Leeds in 1869. At the meeting John Russell Reynolds (1828–96), a physician at University College Hospital who had just relinquished his staff post at the National Hospital for the Paralysed and Epileptic, Queen Square gave a presentation entitled, ‘Paralysis and other disorders of motion and sensation dependent on idea’. Reynolds’ views together with those of Thomas Laycock (1812–76), Crichton-Browne’s teacher at medical school in Edinburgh, who himself had studied at La Pitié hospital in Paris in 1834, had an important lasting influence on Charcot’s views about hysteria. Clifford Allbutt (1836–1925), the most distinguished physician in Leeds at the time of the British Medical Association meeting, had attended the 1861 lectures of Duchenne de Boulogne and Trousseau in Paris and conducted ophthalmoscopic research at the West Riding Asylum. During the meeting there was a delegates’ tour to the West Riding Lunatic Asylum but it is unknown whether Charcot joined the visit or attended the evening party as Crichton-Browne’s guest. The following year, during the siege of Paris by the Germans, the Salpêtrière was shelled and there were epidemics of smallpox, cholera and typhoid in Paris. Charcot continued his work at the hospital but sent his wife and three children to stay in London with their artist friend Ella Casella. Throughout his career Charcot encouraged interaction and collaboration between British and French neurology. He was an honorary member of the British Medical Association and communicated by letter with several British physicians. His example and that of his friend Brown-Séquard encouraged the second generation of physicians at the National Hospital for the Paralysed and Epileptic to become consummate diagnosticians, effective teachers, and also importantly to have a strong commitment to clinical and pathological research.

## Supplementary Material

awaf386_Supplementary_Data
