# Supplementary material for: Charcot redux
Source: Brain. 2025 Oct 14;148(12):4148–54. doi: 10.1093/brain/awaf386 (PMC12677016; doi:10.1093/brain/awaf386)
Supplement: awaf386_Supplementary_Data [file awaf386_supplementary_data.pdf]

Supplementary reading

Pierre Marie and Joseph Babinski. Éloge de J-M Charcot. Revue Neurologique. 1925 6:731-56.

Starr A. Boston Medical History Club. Charcot Centenary Meeting. Boston Medical and Surgical Journal 1926;194:10-20.

Foucault M. The birth of the clinic : an archaeology of medical perception. London: Routledge; 2003. xxii, 266 p. p.

Lees AJ. Brainspotting: Adventures in Neurology. Kendal, England: Notting Hill Edtions 2022.

Goetz CG, Bonduelle M, Gelfand T. Charcot : constructing neurology. New York ; London: Oxford University Press; 1995. xx, 392 : ill. ; 24 cm. p.

Guillain G. J.-M. Charcot, 1825-1893 sa vie, son oeuvre. Paris: Masson; 1955. 1 vol. (188) p.

Charcot JM. Leçons du mardi à la Salpêtrière : policlinique, 1887-1888. Paris: Louis Battaille; 1892. vi, 501 illus. 23 cm. p.

An Occasional Correspondent . Hypnotism in Paris Lancet 1882;120:163-5.

Alvarado CS. Nineteenth Century Suggestion and Magnetism:Hypnosis at the International Congress of Physiological Psychology. Contemporary Hypnosis 2010;27(1):48-60.

Walusinski O. The girls of La Salpetriere. Front Neurol Neurosci. 2014;35:65-77.

Babinski J. Suggestion et hystérie, à propos de l'article de M. Bernheim intitulé 'Comment je comprends le mot hystérie', par J. Babinski. (Extrait du 'Bulletin médical', no 24, 1907.). Chartres, impr. de Durand1907.

Gordon RB. From Charcot to Charlot: Unconscious Imitation and Spectatorship in French Cabaret and Early Cinema. Critical Inquiry. 2001;27(3):515-49.

Baxendale S, Marshall F. The epileptic singers of belle epoque Paris. Med Humanit. 2012;38(2):88-90.

Esparbès Gd, Ibels A, Lefèvre M, Montorgueil G, Ibels H-G. Les demi-cabots, le café-concert, le cirque, les forains dessins de H.-G. Ibels. Paris: G. Charpentier et E. Fasquelle; 1896. III-246 p.

Louis Aragon and André Breton . Le cinquentenaire de l’hysterie. Révolution Surréaliste1928. p. 20-2.

Larner AJ. The West Riding Lunatic Asylum Medical Reports: the precursor of Brain? Brain. 2023;146(11):4437-45.
